# Pulmonary Langerhans cell histiocytosis: the many faces of presentation at initial CT scan

**DOI:** 10.1007/s13244-014-0338-0

**Published:** 2014-07-05

**Authors:** M. C. Castoldi, A. Verrioli, E. De Juli, A. Vanzulli

**Affiliations:** 1 Department of Radiology, Ospedale CTO, via Bignami 1, 20162 Milan, Italy; 2 Department of Diagnostic and Interventional Radiology, Niguarda Ca’ Granda Hospital, Milan, Italy; 3 Department of Pneumology, Regional Reference Center for Rare Lung Disease, Niguarda Ca’ Granda Hospital, Milan, Italy; 4 Department of Diagnostic and Interventional Radiology, Niguarda Ca’ Granda Hospital, Milan, Italy

**Keywords:** Histiocytosis, Interstitial lung disease, Cysts, Nodule, Lung

## Abstract

**Objectives:**

Pulmonary Langerhans cell histiocytosis (PLCH) is a rare interstitial granulomatous disease that usually affects young adults who are smokers. Chest computed tomography (CT) allows a confident diagnosis of PLCH only in typical presentation, when nodules, cavitated nodules and cysts coexist and predominate in the upper and middle lungs.

**Methods:**

This article includes a pictorial essay of typical and atypical presentations of PLCH at initial chest CT. Various appearances of PLCH are illustrated and possible differential diagnosis is discussed.

**Results:**

PLCH can present with some aspecific features that may cause diagnosis of the initial disease to be overlooked or other pulmonary diseases to be suspected. In cases of nodule presentation alone, the main differential diagnosis should include lung metastasis, tuberculosis and other infections, sarcoidosis, silicosis and Wegener’s disease. In cases of cysts alone, the most common diseases to be differentiated are centrilobular emphysema and lymphangiomyomatosis. Clinical symptoms are usually non-specific, although a history of cigarette smoking, coupled with the presence of typical or suggestive findings at imaging, is key to suspecting the disease. Atypical presentations require surgical biopsy for diagnosis.

**Conclusions:**

The radiologist should be familiar with PLCH imaging features to correctly diagnose the disease or need for further investigation.

**Teaching Points:**

• *PLCH is a rare interstitial smoking-related disease that usually affects young adults.*

• *The typical first CT shows a mix of nodules, cavitary nodules and cysts in the upper-middle lungs.*

• *Atypical appearance, either cysts or nodules alone, mandates that other diagnoses be considered.*

• *Lung cystic involvement correlates with lung function abnormalities and predicts functional decline.*

• *Integration of the clinical history and imaging results is key to diagnosis.*

## Introduction

Pulmonary Langerhans cell histiocytosis (PLCH) is an isolated form of Langerhans cell histiocytosis characterised by granulomatous infiltration of the distal bronchial walls with Langerhans cells that produce small nodules and secondarily involve the adjacent arterioles and interstitium. PLCH is rare, representing less than 5 % of all interstitial lung diseases of unknown aetiology [1–3].

PLCH primarily affects young adults, but it can be found across a wide age spectrum, with no gender predilection [2]. It occurs almost exclusively in smokers (90–95 %), which causes its inclusion among smoking-related interstitial diseases [4]. Despite recognition of the promoting role of cigarette smoking, being a smoker rarely induces the disease. This suggests that host-related factors, in addition to an inhaled antigen in cigarette smoke, participate in the pathogenesis of PLCH [5, 6].

Prognosis in PLCH is favourable, yet variable. Most patients experience a clinical and radiological remission (up to 25 %) or stabilisation (up to 50 %). In a minority of patients (15–25 %), the disease progresses to pulmonary failure/cor pulmonale, with end-stage disease characterised by fibrocystic changes that completely replace the lung parenchyma [6, 7].

Lung transplantation is indicated in cases of severe respiratory failure. Death can occur from pulmonary hypertension or respiratory failure. Patient survival at 5 years post-PLCH diagnosis is reported at 87.8 % [8]. Clinical and radiological relapse may occur years after remission [9].

Concerning therapy, based on the evidence associating PLCH with smoking, cessation is believed to promote disease stabilisation and should be taken as the first intervention. Smoking cessation generally results in regression or stabilisation of both symptoms and radiological lesions, yet it cannot be assumed to yield a favourable outcome in any given patient [10, 11]. Corticosteroid use during early disease phases is accepted for control of acute inflammation and constitutional symptoms, but is controversial for overall treatment [12]. Chemotherapy is indicated in patients with multisystem disease or severe isolated pulmonary disease at onset or with progressive worsening of lung function during the disease course [6].

Consequent to disease rarity, there is a lack of longitudinal studies on the long-term natural history of the disease and long-term benefit of therapeutic interventions [7, 13].

## Symptoms, diagnosis, and clinical management at initial patient presentation

**Fig. 1:**
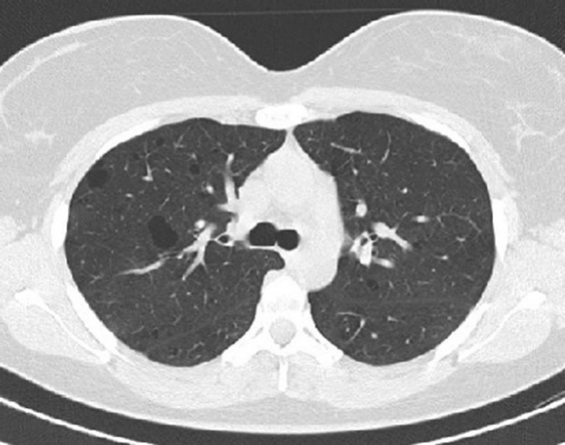
A 20-year-old woman, non-smoker, with PLCH; pulmonary cysts at her first computed tomography (CT) examination. The patient was treated in the neonatal period for a cutaneous form of Langerhans cell histiocytosis that regressed completely during infancy. The cysts are postulated to have resulted from granolomatous transformation of pulmonary histiocytosis, possibly concurrent with the cutaneous paediatric disease. Cysts remained stable during 10 years of follow-up

Upon diagnosis, patients are usually symptomatic with no productive cough and some exertion dyspnea. Pneumothorax can be the first manifestation of the disease in as many as 15 % of patients. Fever and other constitutional symptoms can occur in almost a third of patients. However, PLCH can be incidentally uncovered on radiographs in 25–30 % of cases [2, 5]. The insidious clinical presentation and relatively high percentage of asymptomatic patients can result in a diagnosis overlooked for years. Determination of the actual time of onset can be uncertain; it is even possible that PLCH diagnosed at an adult age may have had its onset in childhood (Fig. 1) [13].

Initial pulmonary function tests (PFTs) can be normal in 10–15 % of patients or demonstrate a mild, predominantly obstructive or mixed pattern [2, 14]. Among PFTs, diffusion capacity (DLCO) is the one most frequently compromised at disease onset. Standard laboratory tests are non-specific and have no diagnostic significance.

PLCH can be strongly suspected on the bases of imaging features. Initial chest radiography can suggest the disease. Thin collimated spiral CT completed with high spatial frequency reconstruction (HRCT) has a high diagnostic accuracy for PLCH when a combination of nodules and cysts is classically shown [15–17]. Nevertheless, because of the myriad of possible CT appearances of PLCH, diagnosis can be challenging, if not impossible, without biopsy.

In adults, bronchoalveolar lavage (BAL) is known to have a lower sensitivity than does parenchimal biopsy, but it is highly indicative of PLCH when the quantity of CD1a-positive cells is >5 % of total cells. Furthermore, it aids the exclusion of other interstitial or infectious lung diseases [2, 6].

Definitive diagnosis is acquired by lung biopsy generally performed by video-assisted thoracoscopic surgery (VATS).

**Fig. 2:**
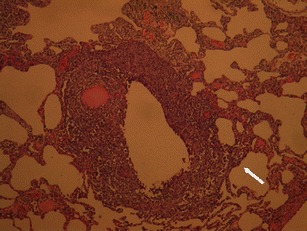
Low-power photomicrograph (original magnification, haematoxylin and eosin stain) of lung tissue shows dense non-necrotising granulomatous infiltration. The sample demonstrates well both localisation of the lesion around a dilated terminal airway and bronchial wall destruction by the infiltrate. The inflammatory cell infiltration also shows an interstitial distribution (*arrow*) (courtesy of Dr. E. Bonacina)

Histopathological diagnosis relies on identification of Langerhans cell (LC) proliferation with terminal and respiratory bronchiole infiltration (Fig. 2) [2, 6, 17]. The abnormal LC proliferation recruits other inflammatory cells to produce brochiolocentric granulomas. Microscopic findings are generally characterised by temporal lesion heterogeneity reflective of the granuloma evolutionary phase, which may transform into a cavitary nodule and then into a thick- and thin-walled cyst. Nodular cavitation reflects airspace dilatation in the centre of granuloma due to rapid destruction of the bronchiole wall by LC infiltration. According to the relative predominance of cellular inflammatory granulomas or fibrotic changes, the early, intermediate and late phases may be distinguished [18].

Also in the late phase of disease, when fibrotic features prevail, LCs may be found in small-sized granulomas harboured in thin-walled cysts [18].

Histological demonstration of the disease is not needed if patients present with mild symptoms, typical features on CT, and require no medical intervention beyond smoking cessation. Nor is biopsy necessary in patients with a histological diagnosis of extra pulmonary disease and findings highly suggestive of PLCH on chest CT.

On the contrary, biopsy is mandatory when CT shows unusual findings, such as if the typical lesion coexistence in different evolutionary phases is absent. Biopsy is also necessary in patients who require immunosuppressive therapy [2, 6].

## Imaging features at initial patient presentation

**Fig. 3:**
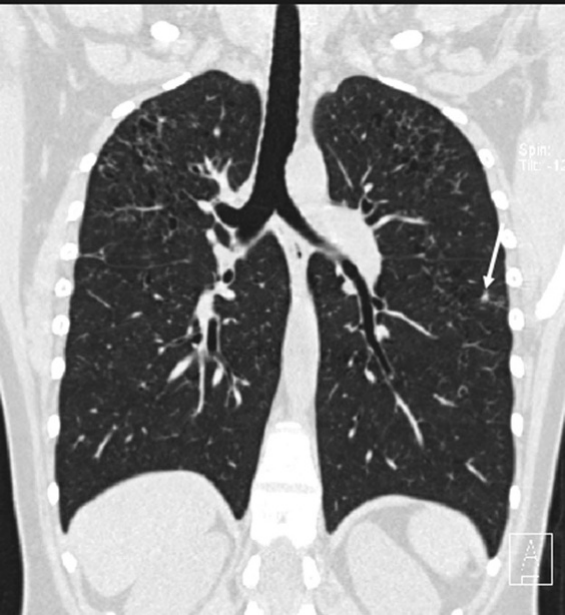
A 41-year-old woman with PLCH. Coronal CT image (thin MIP reconstruction) showing typical distribution of disease: many cysts predominating in upper and middle lungs fiends, with some micronodules (*arrow*); typically, basal regions are spared

The main diagnostic criteria of PLCH include evidence of bronchiolocentric lesions in various features along with their typical distribution pattern that relatively spares basal regions (Fig. 3).

Chest radiography may show ill-defined nodules or reticulonodular changes [16]. Pulmonary alterations are generally symmetric, predominating at the upper-middle lungs and sparing the costophrenic angles. Initial radiographic findings may be just minimally or not at all significant, smoke-related emphysema being the only conspicuous finding.

PLCH appearance on the first CT examination depends on the disease phase [17].

**Fig. 4:**
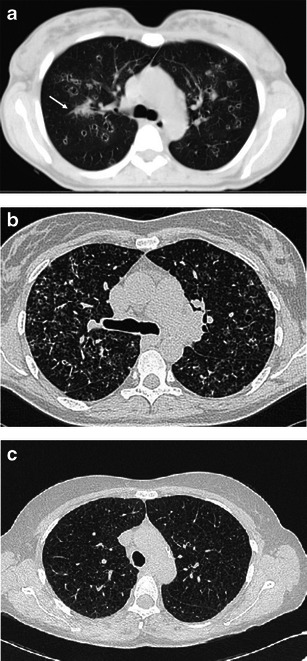
Different imaging presentations of PLCH at clinical onset. Definitive diagnosis obtained by VATS performed within a month of imaging. **a** A 48-year-old woman, smoker, symptomatic for dry cough and mild fever. A 3-mm collimated CT shows a pattern of early “florid” disease characterised by numerous scattered nodules, of which almost all are cavitated and surrounded by ground glass opacities. A consolidation area also coexists (*arrow*). **b** A 46-year-old woman, symptomatic for dry cough and mild fever. HRCT scans show diffuse ill-defined micronodularity, cavitating nodules, cysts and mild interstitial thickening. **c** A 50-year-old woman, smoker, symptomatic for dry cough. HRCT scans show cystic disease with a few scattered micronodules

Early stages present nodular lesions corresponding to “florid” granulomas, while more advanced stages are evidenced by cysts and fibrotic changes (Fig. 4).

Since PLCH may be detected during any of its evolutionary phases, early stage features are more likely, but not exclusively, found at the first CT examination [17, 19].

The early “florid” inflammatory phase is characterised by brochiolocentric, ill-defined micronodules or nodules possibly surrounded by ground-glass opacification secondary to inflammatory interstitial infiltration. Generally, nodules are stellate or peripherically irregular and vary in number and diameter (1–10 mm). In a minority of cases, nodules are larger (>1 cm). Some nodules can present faint lucent centre or be mostly cavitary.

Cysts predominate on nodules in cases in which inflammatory activity is decreased. Thick-walled cysts (>2 mm thick) progressively transform into thin-walled cysts (<2 mm thick).

Cysts may appear round and of small dimension (<1 cm), but in advance disease they are typically larger and of different shapes. Irregular, bi-lobed, cloverleaf, branched or bizarre cysts derive from further cystic dilatation and adjacent cyst fusion. Also, paracicatrical emphysema adjacent to fibrotic changes may appear as cystic spaces.

End-stage disease is demonstrated by a fibrocystic pattern that maintains the typical upper- and middle-lung zone predominance.

**Fig. 5:**
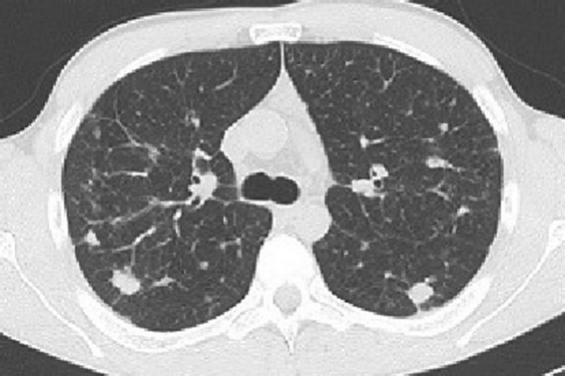
A 34-year-old man, smoker, symptomatic for cough and fever. Definitive diagnosis obtained by VATS performed within a month of imaging. A 3-mm collimated CT shows scattered nodules, with asymmetric distribution. The largest nodules measure 15–20 mm; nodular borders are irregular or atypically smooth. Smooth linear interstitial thickening is also evident

**Fig. 6:**
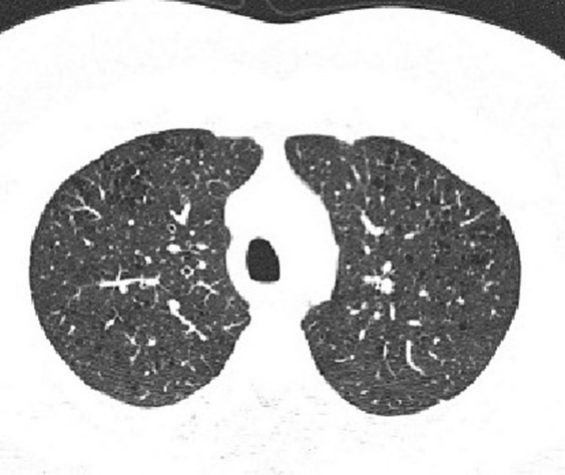
A 38-year-old man, non-smoker, with asymptomatic PLCH and skin involvement. Diagnosis obtained by skin lesion biopsy. BAL >5 % CD1a + cells. HRCT scans at diagnosis show a tiny micronodularity along with multiple areas of centrilobular emphysema

Less common presentation patterns include cases with few, larger than usual, smooth-marginated or highly asymmetrical nodules (Fig. 5). Exceptional imaging presentations, such as alveolar consolidations, single nodules or air-fluid cystic lesions have been reported [20]. Centrilobular emphysema is often found in patients with PLCH, and is likely related to the bronchiolocentric obstructive disease itself, in addition to being a consequence of a smoking habit [21] (Fig. 6). Other findings that can coexist with typical PLCH lesions are those related to smoke exposure, including emphysematous bullae, thickening of the bronchial walls, ground-glass opacities. Smoking related interstitial lung diseases such as respiratory bronchiolitis interstitial lung disease (RB-ILD) and desquamative interstitial pneumonia (DIP) have been described concurrent with PLCH.

## The challenging diagnosis at initial patient presentation

Although the combination of nodules and cysts in various degrees of evolution is a hallmark diagnostic sign of PLCH, the presence of such unique lesions requires that other diagnoses be considered [2, 17]. Table 1 summarises the most common differential diagnosis in case of a unique pattern of lesions presentation.

**Table 1:** Lesion presentation patterns: diseases to differentiate from PLCH

Nodules/micronodules	Cavitating nodules	Cysts
Lung metastasis Tuberculosis/infectious nodules Sarcoidosis, silicosis Wegener disease/vasculitis nodules ^a^RB-ILD	Wegener disease/vasculitis nodules Lung metastasis Septic emboli Cavitated *P. jiroveci* lesions	Lymphangioleiomyomatosis Centrilobular emphysema UIP Lung metastasis LIP^a,b^, HP^a,b^, DIP^a,b^ Cystic fibrohistiocytic tumour Birt Hogg Dubé syndrome Light-chain disease Amyloidosis Pneumatocoeles

^a^*RB-ILD* respiratory bronchiolitis interstitial lung disease, *DIP* desquamative interstitial pneumonia, *LIP* lymphocytic interstitial pneumonia, *HP* hypersensitivity pneumonitis

^b^Usually in association with ground-glass opacities and pulmonary changes that may suggest the disease

### Micronodular and nodular pattern

**Fig. 7:**
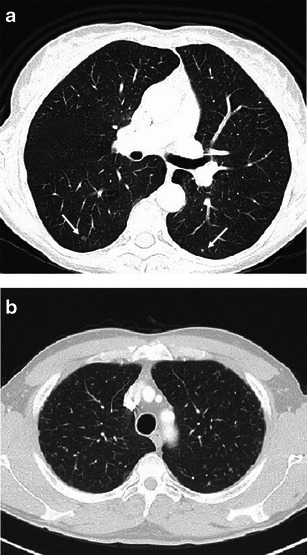
PLCH presenting with nodular features that mimic other diseases. **a** PLCH referred to metastasis: a 44-year-old woman, smoker, with a medical history of carcinoma of cervix uteri, treated with surgery and chemotherapy. A 3-mm collimated CT performed during oncological follow-up shows some faint micronodules in both lungs (*arrows*). Definitive diagnosis of PLCH obtained by VATS. **b** PLCH mimicking sarcoidosis: a 41-year-old man, smoker, with asymptomatic PLCH and skull involvement. Diagnosis obtained by skull biopsy. A 3-mm collimated CT shows numerous millimetric nodules. Nodules are mostly spread at the upper and middle lung fields in a peripheral location; few are subpleuric

In case of exclusively nodular disease, the principal differential diagnoses include lung metastasis, *Mycobacterium* and other infections, silicosis and sarcoidosis, and Wegener’s disease (Fig. 7).

PLCH nodules found during serial imaging examinations in oncologic patients may be indistinguishable from metastasis. In such cases, diagnosis is possible only by biopsy or on retrospective follow-up of the disease evolution [22].

Infectious diseases, presenting with micronodular or nodular patterns may occasionally have PLCH-like features [23]. A thorough medical history, coupled with an assessment of infection risk, may direct the radiologist to the correct diagnosis.

The typical centrilobular distribution of micronodules in PCLH is the main feature to differentiate PLCH from sarcoidosis and silicosis, which also predominate at the upper regions but are characterised by a prominent perilymphatic distribution of nodules. Nevertheless, in isolated nodules, this distinctive feature can be missed due to granuloma coalescence [24]. Another diagnostic differential criterion is the absence of mediastinal and hilar lymph nodes in PLCH.

**Fig. 8:**
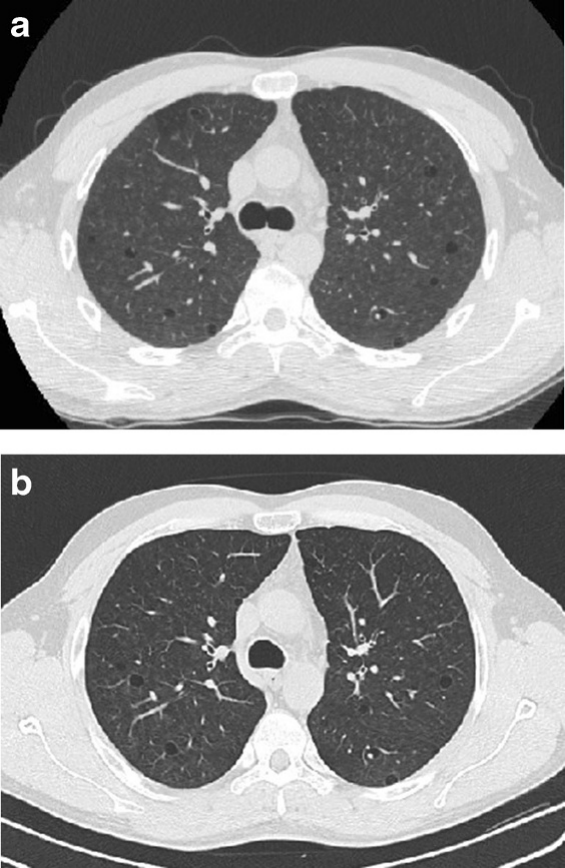
A 35-year-old man, smoker, with asymptomatic PLCH (chest radiograph performed for traumatic injury). Diagnosis based on typical lesion heterogeneity on CT imaging. **a** Two-millimetre collimated CT images at initial diagnosis shows bilateral centrilobular ground-glass nodules, thin-walled cysts and a single air-trapping zone. Upper and middle lung are predominantly affected. **b** Three-millimetre collimated CT after 5 months of smoking cessation shows almost complete resolution of the centrilobular nodularity while thin-walled cysts remain unchanged

Early PLCH can have the same features as RB-ILD, presenting as bronchiolocentric ill-defined micronodules generally associated with mild centrilobular emphysema (Fig. 8). Fortunately, clinical management of both diseases is similar in that smoking cessation is the most important medical intervention.

### Nodular cavitary pattern

**Fig. 9:**
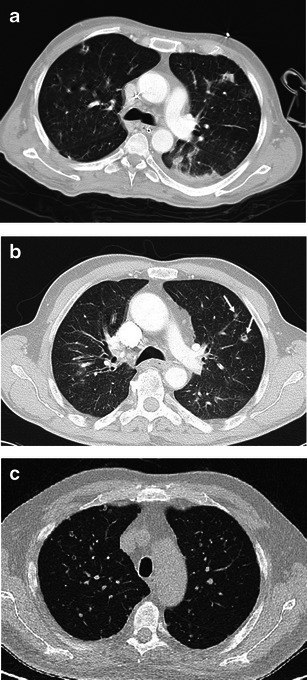
Diseases requiring differentiation from PLCH when cavitary nodules are the main presenting feature. **a** Septic pulmonary embolism in a critically ill patient with endocarditis. A 3-mm collimated CT scan demonstrates both a few solid and cavitary scattered nodules, compatible with haematogenous seeding; some consolidative opacities are also present on the left lung. **b** Biopsy-confirmed Wegener’s disease in a 67-year-old man with systemic symptoms and dry cough. A 3-mm CT scan shows two pulmonary nodules (*arrows*), one of which is cavitated, bilateral bronchiectatic changes and an ill-defined ground glass opacity in the right lung. **c** Cavitating metastasis in a 68-year-old man with hilar cholangiocarcinoma at onset. A 3-mm collimated CT scan reveals numerous solid and cavitary small nodules, showing relatively thin walls

When cavitary nodules are prominent, the main differential diagnoses include Wegener’s granulomatosis, septic and metastatic nodules, cavitated *Pneumocystis jiroveci* lesions (Fig. 9).

Wegener’s granulomatosis, which is also called granulomatosis with polyangiitis, is a necrotising vasculitis that affects small- to medium-sized vessels and commonly involves the lungs, the kidneys and upper respiratory tract. Among the multiple presentation features, Wegener’s disease can appear as a few, randomly distributed pulmonary nodules or, less commonly, as centrilobular micronodularity [25]. Nodules cavitate in up to 50 % of cases due to primary collagen necrosis, which can make the nodules of Wegener’s granulomatosis look similar to those in the “florid” phase of PLCH. A wide spectrum of laboratory abnormalities is usually found in Wegener’s disease.

Metastatic nodular cavitation may occur in the squamous cell type, although nodules from adenocarcinomas and sarcomas may infrequently do so [26]. Cavitary metastases generally display an irregular thickening of the walls, although thin-walled nodules can be found.

Septic emboli or excavated pneumocystosis may appear indistinguishable from PLCH nodules on CT imaging. Differential diagnosis requires consideration when a patient is at-risk or when constitutional symptoms are present. In either event, confirmation by BAL or biopsy is needed.

### Cystic pattern

The most common differential diagnoses in predominantly cystic PLCH include usual interstitial pneumonia (UIP), centrilobular emphysema, and lymphangioleiomyomatosis (LAM) [27–29].

Diagnostic problems usually do not subsist in UIP due to the typical laterobasal abnormalities distribution, layered arrangement of cysts, intralobular interstitial thickening and traction bronchiectasis.

**Fig. 10:**
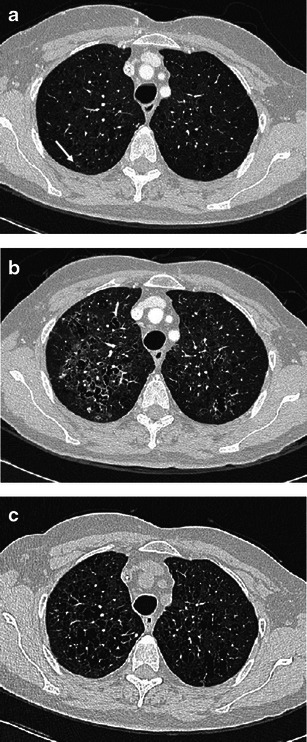
A 68-year-old woman, smoker, symptomatic with excertional dyspnea. Definitive diagnosis obtained by VATS performed at time of **b**. **a** A 1-mm collimated CT shows focal air spaces that appear as emphysema. A faint micronodule is also present (*arrow*). **b** A 1-mm collimated CT 9 months after **a** with no smoking cessation demonstrates transformation and progression of focal air spaces that now appear as irregular cystic lesions surrounded by ground-glass opacities. Some scattered nodules coexist. **c** At 3-year follow-up, HRCT scans show cystic disease progression with some non-walled cysts. Parenchymal infiltrates have regressed

Centrilobular emphysema typically appears as lucent areas lacking distinct walls with a central centrilobular artery. Occasionally, however, centrilobular emphysema may show partial walls related to fibrotic peripheral changes, and vice versa, the cyst walls of pulmonary histiocytosis may appear so thin as to simulate emphysema (Fig. 10).

LAM affects pre-menopausal women in a sporadic form or, most commonly, with tuberous sclerosis complex. It is a pure cystic disease, cysts being caused by air trapping mechanism due to smooth-muscle cell peribronchial proliferation [30]. Pneumothorax is the first manifestation of LAM in up to 50 % of patients.

**Fig. 11:**
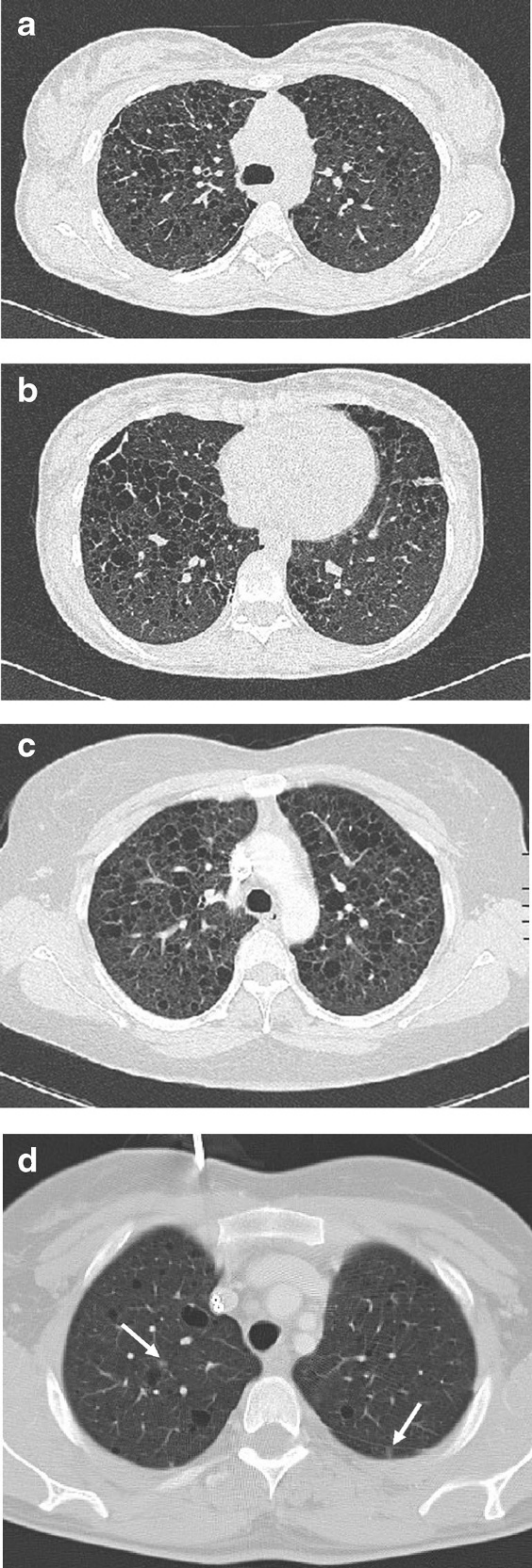
Cystic PLCH compared with LAM **a**, **b** A 22-year-old woman hospitalised for pneumothorax. Medical history was negative for respiratory disease. Definitive diagnosis of PLCH obtained by VATS performed within a month of imaging. HRCT scans show numerous thin-walled cysts of mostly ovoid and polygonal shape with a nearly imperceptible predominant craniocaudal distribution. Persistence of a small pneumothorax is shown. **c**, **d** Biopsy-confirmed LAM in two young female patients. A 3-mm collimated CT scan shows diffuse (**c**) and mild (**d**) pulmonary involvement of thin-walled cysts randomly distributed throughout both lungs, with some faint micronodules (*arrows*) (compare **d** with Fig. 1)

As in PLCH, HRCT highlights thin-walled cysts. Three features can help to distinguish LAM from PLCH: cysts have a more regular morphology and lack the bizarre shapes typical of advanced PLCH; cysts are randomly distributed without sparing the costophrenic angles; parenchymal nodules are less frequent, although reported in a small number of cases. Despite these differences, pulmonary findings can be confounding between the two diseases, such that biopsy is mandatory in young females with a pure cystic pattern at onset/initial presentation (Fig. 11).

The cysts of PLCH should be differentiated from the pneumatocoeles which accompany primary pulmonary infections (such as *Staphylococcus aureus* and *P. jiroveci*) and that usually resolve with treatment of the underlying infection.

Cystic PLCH must also be differentiated from rare cystic lung diseases, which are mostly associated with peculiar clinical presentations [27, 28, 31]. Lung cysts found in amyloidosis and light-chain deposition disease (LCDD) generally occur in association with multiple myeloma or macroglobulinaemia. In inherited Birt-Hogg-Dubé syndrome, thin walled pulmonary cysts are characteristically found in association with facial papules and an increased risk of developing renal tumours [31] .

Rarely, pulmonary metastasis can be pure cystic in tumours of epithelial origin and, less frequently, in sarcomas. Moreover, cystic PLCH must be differentiated from the rare cystic fibrohistiocytic tumour, both in the primary and in the secondary forms. Pulmonary cysts of this tumour also result from pulmonary nodule transformation [32]. Histological assessment is required for diagnosis.

Scattered cysts can be found in several interstitial lung diseases (ILDs) like lymphocytic interstitial pneumonia (LIP), hypersensitivity pneumonitis (HP) and desquamative interstitial pneumonia (DIP). Diagnosis of these diseases is generally suggested by the typical association of cysts with ground-glass opacities and characteristic pulmonary changes. Moreover, LIP usually occurs with lympho-proliferative or autoimmune disorders, typically representing the most common lung pathology in Sjogren syndrome.

### Reticular pattern

**Fig. 12:**
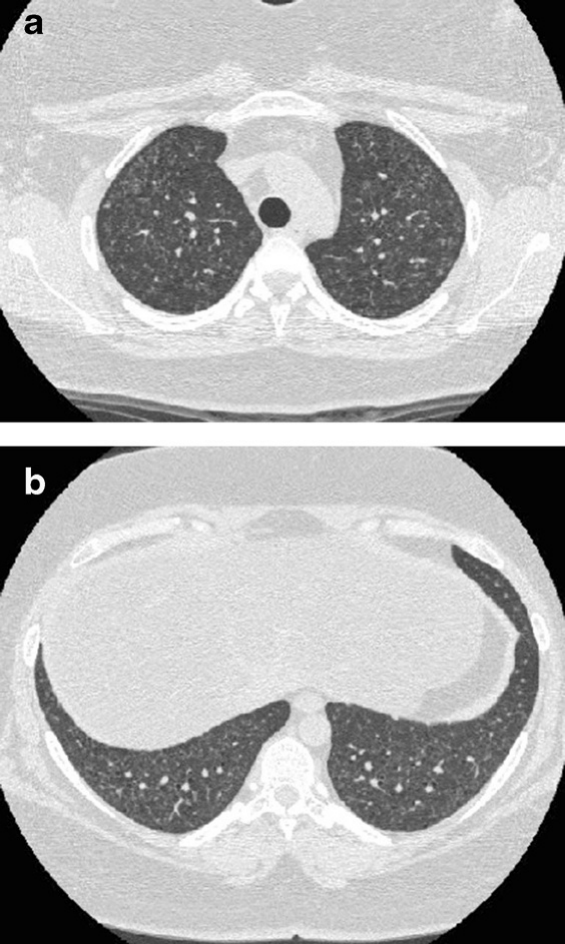
A 34-year-old woman non-smoker with PLCH symptomatic for dry cough. Definitive diagnosis obtained by VATS performed within a month of imaging studies. **a**, **b** HRCT images at diagnosis shows ill-defined micronodularity associated with predominant reticular and minimal bronchiectatic changes. No basal regions sparing

PLCH may rarely present prominent reticular interstitial changes that appear as multiple interlacing lines in a net-like arrangement [18]. Linear opacities are fine thickness and generally coexist with a few small nodular or cystic changes. In these cases, diagnosis of PLCH require careful search of the characteristic lesions of the disease (Fig. 12).

## Prognostic implication of first CT imaging

**Fig. 13:**
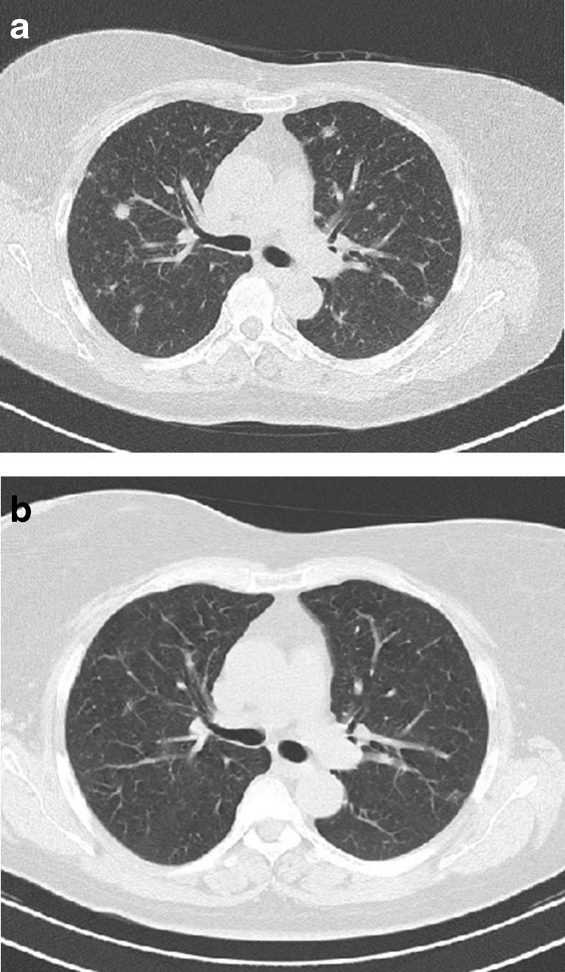
A 61-year-old woman, smoker, with asymptomatic PLCH and bone involvement. Diagnosis obtained by scapular biopsy. **a** A 3-mm collimated CT demonstrates nodular infiltrates with irregular borders. Some minimal faint cysts are also appreciable (*arrow*). **b** A follow-up CT scan 6 months post-diagnosis shows almost complete regression of pulmonary nodules. Improvement occurs with smoking reduction, but not cessation

A strong correlation exists between disease histopathological inflammatory activity and the different phase patterns shown by CT imaging. The early “florid” phase corresponds to the most active inflammation and has potential for regression (Fig. 13). In fact, solid and cavitary nodules may regress or resolve completely, while cysts infrequently show improvement on follow-up CT studies [33]. Generally, recurrent pneumothorax implies a negative evolution of the disease [2].

The extent of lung cystic involvement at first CT examination correlates with pulmonary function abnormalities at presentation and has a predictive value for later development of functional impairment. Specifically, severe cystic changes at onset correlate with risk of a rapid decline until eventual respiratory insufficiency [14, 34].

Patients with a predominant cystic pattern at onset are still believed to benefit from long-term follow-up, although the utility of serial CT during the course of the disease is presently debated [14, 17, 34]. Functional tests seem to be better predictors of prognosis relative to imaging [35]. In fact, cyst progression shown by follow-up CT studies is associated, but does not anticipate, the decline of functional tests [36].

Both improvement or progression of pulmonary lesions occur during a few months (14–22 m).

## Conclusions

PLCH is a rare disease whose elementary lesion is a bronchiolocentric-evolving granuloma. A history of cigarette smoking, coupled with suggestive findings at radiographic imaging, is key to suspect the disease. At diagnosis, symptoms and functional tests are non-specific, while CT may be distinctive, showing lesions corresponding to the various evolutionary aspects of the disease. Typical cases are characterised by the association of nodules, cavitary nodules and cystic lesions. However, differential diagnosis can be challenging when CT shows unique lesions that can mimic other diseases.

Knowledge of the different CT features at presentation helps to determine the diagnosis of PLCH correctly or to consider PLCH among other diseases and thus guide the diagnostic approach.
